# The Simple Triage Scoring System (STSS) successfully predicts mortality and critical care resource utilization in H1N1 pandemic flu: a retrospective analysis

**DOI:** 10.1186/cc10001

**Published:** 2011-01-26

**Authors:** Kayode A Adeniji, Rebecca Cusack

**Affiliations:** 1 Critical Care Research Unit, SUHT, Southampton General Hospital, Tremona Road, Southampton, SO16 6YD, UK

## Abstract

**Introduction:**

Triage protocols are only initiated when it is apparent that resource deficits will occur across a broad geographical area despite efforts to expand or acquire additional capacity. Prior to the pandemic the UK Department of Health (DOH) recommended the use of a staged triage plan incorporating Sepsis-related Organ Failure Assessment (SOFA) developed by the Ontario Ministry of Health to assist in the triage of critical care admissions and discharges during an influenza outbreak in the UK. There are data to suggest that had it been used in the recent H1N1 pandemic it may have led to inappropriate limitation of therapy if surge capacity had been overwhelmed.

**Methods:**

We retrospectively reviewed the performance of the Simple Triage Scoring System (STSS) as an indicator of the utilization of hospital resources in adult patients with confirmed H1N1 admitted to a university teaching hospital. Our aim was to compare it against the staged initial SOFA score process with regards to mortality, need for intensive care admission and requirement for mechanical ventilation and assess its validity.

**Results:**

Over an 8 month period, 62 patients with confirmed H1N1 were admitted. Forty (65%) had documented comorbidities and 27 (44%) had pneumonic changes on their admission CXR. Nineteen (31%) were admitted to the intensive care unit where 5 (26%) required mechanical ventilation (MV). There were 3 deaths. The STSS group categorization demonstrated a better discriminating accuracy in predicting critical care resource usage with a receiver operating characteristic area under the curve (95% confidence interval) for ICU admission of 0.88 (0.78-0.98) and need for MV of 0.91 (0.83-0.99). This compared to the staged SOFA score of 0.77 (0.65-0.89) and 0.87 (0.72-1.00) respectively. Low mortality rates limited analysis on survival predictions.

**Conclusions:**

The STSS accurately risk stratified patients in this cohort according to their risk of death and predicted the likelihood of admission to critical care and the requirement for MV. Its single point in time, accuracy and easily collected component variables commend it as an alternative reproducible system to facilitate the triage and treatment of patients in any future influenza pandemic.

## Introduction

The word 'triage' originates from the French 'trier' (to choose from among several) and was originally applied around 1792 by Baron Dominique Jean Larrey, surgeon in chief to Napoleon's Imperial Guard, as a process of sorting wounded soldiers. Its aim was to optimize the use of available medical resources to maximize efficacy [1]. Patients with the greatest chance of survival with the least resource use are treated first [2]. In disaster situations, the focus of medical care is directed toward the needs of the community. In this approach, it is clear that the standard of care for all patients, including those not directly related to the incident, may need to be adjusted and reduced. While this may infringe on individual rights, the higher ethical principle of 'wellness of society as a whole' calls for the direction of resources to those for whom it is felt to be the most effective.

An influenza pandemic had been expected for a number of years. The resulting preparations led to the need to examine how different health-care systems around the world could respond to such an event, which may require a large surge in the need for critical care capacity. On 11 June 2009, the World Health Organization declared the first influenza pandemic since 1968. Emerging from a triple-reasortant virus circulating in North American swine [3], the new influenza A virus variant, H1N1, has affected more than 213 countries and territories worldwide [4]. Epidemiological models assumed that the peak demand for critical care resources would significantly outstrip supply [5]. The pandemic declaration brought into sharp focus the strategic planning that the international community had been developing since the outbreak of avian flu H5N1 in 2005 [6]. Surge capacity planning identified the need for a consistent and objective triage system that was based on physiological scores and that was valid, reproducible, and transparent given the likelihood of a need to ration critical care resources [4].

The UK Department of Health (DOH) recommended a system devised by a panel of experts commissioned by the Ontario Ministry of Health. This system was proposed to guide 'critical care resource allocation issues' during the initial days and weeks of an overwhelming influenza pandemic and to prioritize admission to critical care beds. After an exhaustive literature search, the Sepsis-related Organ Failure Assessment (SOFA) score [7] was suggested as part of a staged triage and treatment prioritization tool to be used in association with a number of inclusion and exclusion criteria based upon comorbidities and estimated prognosis [8,9].

The SOFA score has been shown to reliably evaluate and quantify the degree of organ dysfunction present on admission to the intensive care unit (ICU) (initial score) or developing during ICU stay (delta score equals subsequent total maximum SOFA scores minus admission total SOFA). The maximum SOFA score reflects cumulative organ dysfunction that develops and correlates with mortality, whereas the mean score is a good prognostic indicator predicting outcome throughout an ICU stay [10,11].

The Ontario Working Group's proposal to aid mass triage had no information on the epidemiology or pathophysiology of the virus that would cause the pandemic. The clinical course of H1N1 pandemic influenza has not occurred as expected. As data has become accessible, reports suggest that SOFA score may not be a good discriminator of outcome in this cohort of patients [12,13]. Thus, its suitability as a means to assist in the triage of H1N1 patients has been called into question.

The Simple Triage Scoring System (STSS) (Table 1), which uses only those vital signs and patient characteristics that are readily available at initial presentation, was proposed in 2007 by Talmor and colleagues [14] as a potential alternative tool in predicting death and the utilization of critical care resources during epidemics. Its components are age, shock index (heart rate > blood pressure), respiratory rate, oxygen saturation, and altered mental state. In a multicenter retrospective analysis of prospectively collected data, the STSS score variables were validated in two cohorts of patients (*n *= 1,927) presenting with sepsis to one of two emergency departments (EDs). The score was found to be predictive of the need for admission to the ICU and the requirement for mechanical ventilation (MV) and of the primary outcome of mortality. Our objective was to review the performance of the admission STSS and SOFA scoring systems as indicators of the utilization of hospital resources and of mortality in H1N1 infection patients admitted to a UK university teaching hospital.

**Table 1:** The Simple Triage Scoring System

Variable	Odds ratio	95% confidence interval	Complex rule points	Simplified (final) rule points
Respiratory rate >30 breaths per minute	3.9	2.5-6.3	4	1
Shock index >1 (HR > BP)	2.8	1.8-4.2	3	1
Low oxygen saturation	2.8	1.8-4.2	3	1
Altered mental status	1.9	1.3-2.8	2	1
Age of 65 to 74 years	3.0	1.7-5.5	3	1
Age of at least 75 years	4.4	2.7-7.2	4	1

BP, blood pressure; HR, heart rate. Reproduced with the kind permission of Wolters Kluwer Health [14].

## Materials and methods

In a service evaluation assessment, we retrospectively reviewed the records of all adult patients who were admitted to the hospital and who were subsequently confirmed to have contracted H1N1 between July 2009 and February 2010. The study was conducted under the auspices of the Critical Care Department Research Unit of the Southampton University Hospital Trust. Pertinent demographic data, comorbidity, initial chest x-ray (CXR) findings, mode of ventilatory support, level of care, bed days, mortality, and the physiological and laboratory components required to calculate the STSS and SOFA scores at the point of hospital admission were collected. Where an arterial blood gas result was not available to calculate the respiratory component of the SOFA score, the validated oxygen saturation as measured by pulse oximetry/fraction of inspired oxygen (SpO_2_/FiO_2_) ratio correlations derived by Pandharipande and colleagues [15] were used. Our institution's pandemic flu protocol called for the involvement of critical care in any patient whose FiO_2 _requirements exceeded 60% to maintain an arterial partial pressure of oxygen (PaO_2_) of greater than 8 kPa. The discriminatory power of the individual score groupings was calculated and analyzed to assess their performance in the initial triage of the H1N1 patient with reference to mortality (primary outcome) and the need for ICU and need for MV (secondary outcomes).

### Statistics

The accuracy of each score in predicting outcome was assessed by plotting the receiver operating characteristic (ROC) curve and calculating the area under the ROC curve (AUC) with 95% confidence intervals [16]. The AUC values were 'ranked' as excellent (AUC of 0.90 and above), good (AUC of from 0.80 to less than 0.90), fair (AUC of from 0.70 to less than 0.80), and poor (AUC of less than 0.70) (SPSS Statistics 19 Inc., Chicago, IL, USA, and IBM, Armonk, NY, USA).

## Results

Sixty-two adult patients (35 males) were admitted to our hospital from either the medical assessment unit or ED with a polymerase chain reaction-confirmed diagnosis of H1N1. Their mean age (range) was 41 (18 to 71) years (Table 2). Forty (65%) had either one (32) or two (8) comorbidities documented (25 respiratory), three were morbidly obese, and three were pregnant. Twenty-seven (44%) had either a secondary bronchopneumonia or lobar pneumonia reported and formally confirmed by a consultant radiologist on the admission CXR. Nineteen (31%) were admitted to the ICU, where three required only supplementary oxygen, 11 (58%) were managed with noninvasive ventilation (NIV), and five (26%) required intubation and MV.

**Table 2:** Clinical characteristics of H1N1 patients admitted to the hospital

Patient characteristics	Ward-based	ICU admissions
Number of patients	43	19
Age in years, median (range)	35 (19-71)	53 (18-71)
Sex		
Male	26	9
Female	17	10
Comorbidity		
1	20	12
2	3	5
≥3	0	0
Obese	0	3
Pregnant	3	0
Abnormal chest x-ray on admission		
Bronchopneumonia	10	9
Lobar pneumonia	4	3
Pulmonary edema	0	1
Mode of ventilation: NIV/MV	0/0	11/5
Hospital outcomes		
Bed days, median (range)	4 (0-13)	7 (1-46)
Mortality	0	3^a^

^a^One patient was transferred from the intensive care unit (ICU) to the ward for palliation. MV, mechanical ventilation; NIV, noninvasive ventilation.

There were three deaths, all with pneumonic features on their admission CXRs, and two were invasively ventilated. Of the two male patients with an STSS score of 2, one had chronic obstructive pulmonary disease (COPD) and the other had COPD and biventricular failure. The latter patient had been treated for H1N1 a week previously and discharged home and re-presented with a secondary bacterial chest infection and associated sepsis. He was still H1N1-positive at this time. He suffered a myocardial infarction and went into multiple organ failure. The patient with an STSS score of at least 3 was an 18-year-old female with no comorbidities, presented after being symptomatic for 5 days with bilateral bronchopneumonic changes on her CXR, and died from complications of extracorporeal membrane oxygenation (ECMO) at the national ECMO center.

The median time from presentation to admission to the ICU for 16 out of 19 (84%) patients was less than 24 hours (range of 0 to 2 days). Of the three remaining patients, two were admitted to the ICU after 24 hours as in-patients and had STSS scores of 2 and SOFA scores of 3 and 5, respectively. The third patient was admitted to the ICU 48 hours after hospital admission and had an STSS score of 2 and a SOFA score of 1; that patient did not survive. The median (range) numbers of level 2 and level 3 critical care bed days used for H1N1 patients over the study period were 4 (2 to 23) and 23 (1 to 46) days, respectively.

**Figure 1:**
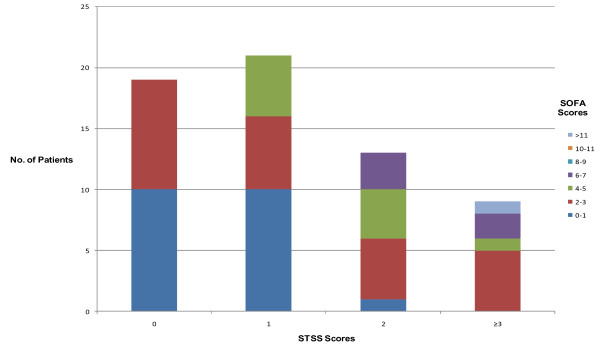
**Graph comparing the calculated Simple Triage Scoring System (STSS) with the Sepsis-related Organ Failure Assessment (SOFA) score patient categories**.

A comparison of the STSS and SOFA score categorization shows a reasonable agreement regarding the severity of the patients' illness (Figure 1). The admission STSS (Table 3) and initial SOFA (Table 4) scores were calculated and compared against actual mortality, need for ICU admission, and need for MV. The performance of STSS and SOFA in our subset is compared with the figures quoted in the original reports [9,13], in which the ROC AUC results were used to assess the performance of the individual score groupings.

**Table 3:** Discrimination of STSS score groupings in predicting death, ICU admission, and need for mechanical ventilation in our study population (*n *= 62) and the derivation population (*n *= 3,206)

STSS score	**Mortality**, fraction (percentage)		Need for ICU			**Need for MV**, fraction (percentage)	
			
	Derivation group^a^ *n *= 3,206	Study group *n *= 62	Derivation group^a^, fraction (percentage)	**Study group**, fraction (percentage)	Study bed days, median (range)	Derivation group^a^	Study group
0	5/1,144 (0.4)	0/19 (0)	61/1,144 (5.3)	1/19 (5.3)	4	18/1,144 (1.6)	0/19 (0)
1	45/1,257 (3.6)	0/21 (0)	124/1,257 (9.9)	2/21 (9.5)	3 (2-4)	37/1,257 (2.9)	0/21 (0)
2	54/617 (8.8)	2/13 (15.3)	140/617 (23)	7/13 (53.8)	9 (2-46)	43/617 (7)	1/13 (7.7)
≥ 3	47/188 (25)	1/9 (11.1)	68/188 (36)	8/9 (88.8)	8 (3-24)	25/188 (13)	4/9 (44.4)
ROC AUC (95% CI)	0.8	Sample too small	0.7	0.88 (0.78-0.98)^b^	-	0.69	0.91 (0.83-0.99)^b^

^a^Talmor and colleagues [14]; ^b^Figure 2: Receiver operating characteristic (ROC) curves for Simple Triage Scoring System (STSS) versus intensive care unit (ICU) and STSS versus mechanical ventilation (MV). AUC, area under the curve; CI, confidence interval.

**Table 4:** Discrimination of initial SOFA score groupings in predicting mortality in our study population (*n *= 62) and in the derivation population (*n *= 352)

Initial SOFA Score	**Mortality**, fraction (percentage)		Need for ICU		Need for MV, fraction (percentage)
			
	Derivation group^a^ *n *= 352	Study group *n *= 62	**Study group**, fraction (percentage)	Study bed days, median (range)	Study group
0-1	0/43 (0)	1/21 (4.8)	2/21 (9.5)	10.5 (4-17)	0/21 (0)
2-3	5/77 (6.5)	1/25 (4)	9/25 (36)	4 (2-23)	2/25 (8)
4-5	18/89 (20.2)	0/10 (0)	2/10 (20)	15 (8-22)	0/10 (0)
6-7	14/65 (21.5)	1/5 (20)	5/5 (100)	4 (1-46)	2/5 (40)
8-9	11/33 (33.3)	0/0 (0)	0/0 (0)	0	0/0 (0)
10-11	12/24 (50)	0/0 (0)	0/0 (0)	0	0/0 (0)
>11	20/21 (95.2)	0/1 (0)	1/1 (100)	24	1/1 (100)
ROC AUC (95% CI)	0.79	Sample too small	0.77 (0.65-0.89)^b^	-	0.87 (0.72-1.00)^b^
>11 and exclusion criteria	20/21 (95.2)	0/5 (0)	3/5 (60)	-	2/5 (40)

**Figure 2:**
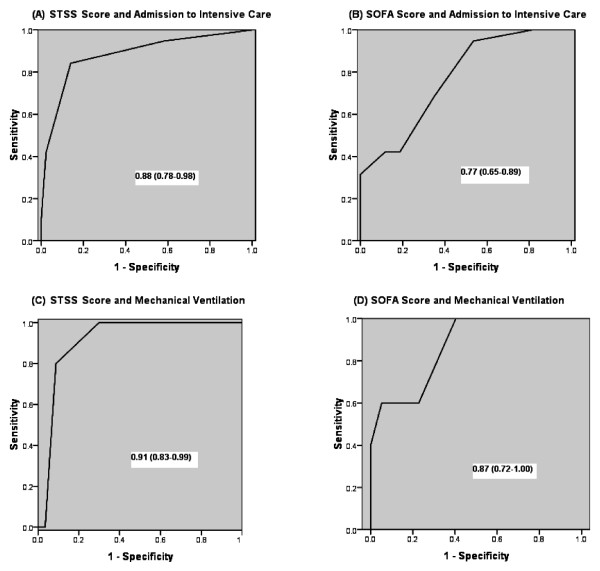
**Comparisons of the area under the receiver operating characteristic curves predicting admission to the intensive care unit and requirement for mechanical ventilation**. (A) STSS scores ability to predict the H1N1 patients admission to the ICU. (B) SOFA scores ability to predict the H1N1 patients admission to the ICU. (C) STSS scores ability to predict the H1N1 patients subsequent need for mechanical ventilation. (D) SOFA scores ability to predict the H1N1 patients subsequent need for mechanical ventilation. SOFA, Sepsis-related Organ Failure Assessment; STSS, Simple Triage Scoring System.

These data were used to assess whether Sepsis-related Organ Failure Assessment (SOFA) score had some functionality in predicting need for intensive care unit (ICU) and need for mechanical ventilation (MV) in our study group. ^a^Ferreira and colleagues [10]; ^b^Figure 2: Receiver operating characteristic (ROC) curves for SOFA versus ICU and SOFA versus MV. AUC, area under the curve; CI, confidence interval.

Owing to the low H1N1-related mortality rate in our cohort, analysis of this outcome data was not possible. However, the trends suggest that a higher STSS score equates with higher mortality in comparison with a high SOFA score. The SOFA score performed well in predicting the need for admission to the ICU (ROC AUC 0.77; 0.65 to 0.89) and the requirement for MV (ROC AUC 0.87; 0.72 to 1.00). Nonetheless, the performance of the STSS score was better at predicting need for ICU admission (ROC AUC 0.88; 0.78 to 0.98) and need for MV (0.91; 0.83 to 0.99) (Figure 2).

If the exclusion criteria within the staged triage protocol that incorporated SOFA (developed by Ontario and recommended by the UK DOH) - that is, SOFA score of greater than 11, chronic lymphocytic leukemia (after allogenic transplant), lymphoma (after allogenic transplant), cervical cancer, and cystic fibrosis - had been applied, a total of five patients would have been excluded from ICU admission. All of these patients survived this hospital admission to discharge.

## Discussion

During a mass casualty event, the triage of patients to determine those who may require, do require, and are receiving definitive critical care interventions needs continuous re-evaluation to impartially allocate the limited resources. Once surge capacity has reached its limit, the delivery of critical care moves to the 'process of last resort' (that is, triage) [17]. Ideally, the selected prioritization tool needs not only to be able to facilitate tertiary critical care triage (the allocation of mechanical ventilators) [18] but also to be applied in the community, assisting general practitioners in deciding which patients should be referred to the hospital and assisting hospital physicians in deciding whether referral to the ICU is appropriate [19].

Recently, there has been a great effort to define these prerequisite basic concepts and assumptions to cope with critical care resource allocation with many references to military experience. Standard operating procedures describing the rationale, components, implementation, and framework to guide and support the development of local and national protocols, particularly with regard to mass infection, have been published [20-22].

A decision matrix in which the weight of objective prognostic information supersedes any subjective or individual patient factors may be an uncomfortable paradigm of deliberation for the physician. Therefore, to ensure justice both to the physician and to the patient, there has to be institutional oversight and 'guidelines'. A triage plan will equitably provide every person the opportunity to survive but cannot guarantee either treatment or survival [8]. However, such a plan cannot supersede the judgment of a physician faced with the triage situation as it is only upon retrospective analysis of the true outcomes of individual patients compared with their predicted outcomes and triaged status that an evaluation of the appropriateness and justice of the triage decision can be made [2].

The SOFA score was originally designed to describe the morbidity ensuing from organ dysfunction in critically ill patients over the course of their ICU stay. The maximum SOFA score and the delta SOFA have been shown to be good instruments in the evaluation and quantification of the degree of organ dysfunction/failure present on admission to the ICU [11]. Ferreira and colleagues [10] evaluated the initial, mean, highest, and delta SOFA scores in a cohort of 352 patients admitted to their ICU in Belgium. Scores were then correlated with mortality. The authors showed that an initial SOFA score of up to 9 predicted a mortality of less than 33% whereas a score of greater than 11 predicted a mortality of 95%. When the initial score was 8 to 11, an unchanged or increasing score was associated with a mortality rate of 60% (initial score of 2 to 7 and mortality of 37%). Therefore, a positive delta SOFA score during the first 48 hours of an ICU admission predicted a mortality of at least 50%.

The Ontario protocol, as in the military, advocated the application of a color-coded prioritization tool. Reassessment would occur at specified time periods (approximately 48 and 120 hours) by a member of the triage team. Patients not meeting the inclusion criteria at reassessment or deteriorating and not expected to survive are transferred to the ward for ongoing care or palliation, respectively. This re-evaluation would also be applied to ward-based patients who deteriorate or improve. The 'cutoff' would be the presentation with or development of a SOFA score of greater than 11 [2,8,19,20,22].

Predictive validity considers the degree to which the triage acuity level predicts true acuity. The primary problem with predictive scoring systems is that they are population-specific and are derived from and validated on specific cohorts of patients and thus their ability to predict the outcome of an individual is poor [23]. Therefore, the decision to reassign a mechanical ventilator from one patient to another would be difficult to justify unless a large difference (approximately 25%) [22] in the survival advantage predicted by the scoring system were demonstrated. Critical care scoring systems have not been designed for this purpose.

In this study, we assessed the performance of an alternative scoring system, the STSS, in H1N1 patients admitted to a UK teaching hospital. We compared this with the initial SOFA score as the organ dysfunction measure within a triage prioritization tool. To our knowledge, the performance of STSS has not been tested on patients during a pandemic. Although the STSS was developed in a cohort of individuals presenting with a variety of infectious diseases to an ED, it was recommended for use during a pandemic. It is simple and can be carried out by a wide range of health-care staff in a variety of settings. Importantly, the STSS score excludes both the patient's history (which may not be available at the point of entrance into the medical system) and laboratory testing (which adds time delay to triage). SOFA incorporation of the latter would result in a delay in decision making and consumption of stretched laboratory resources. Additionally, whereas the variability in the subjective assessment of the Glasgow Coma Scale component in the SOFA may affect its inter-observer accuracy, the binary nature of the comparison measure in the STSS of 'altered mental state' may ameliorate this source of error. In our cohort of patients, STSS performed well, risk-stratifying patients with this viral illness with regard to the need for admission to the ICU and need for MV.

The clinical nature of any pandemic becomes apparent only as the event unfolds. Fortunately, the mortality of H1N1 has been considerably less than that seen with H5N1. It is widely reported that the clinical attack rate and most severe disease have been highest in the young [24]. The Australasian and Canadian experiences of the 2009 pandemic demonstrated how population differences in different communities can confound the application of 'evidence' across these disparate populations. Kumar and colleagues [25] reported that 43/168 (25.6%) of critically ill flu patients in Canada over the period described were Inuit, who represent just 3.75% of the population. Similarly, the ANZIC (Australian and New Zealand Intensive Care) investigators reported that the Aboriginals (2.5% of the population) represented 9.7% of the ICU admissions in Australia and the Maoris (13.6% of the population) emerged as 25% of the critically ill in New Zealand [25,26]. The pattern of illness was markedly different in our population. Without a native indigenous population that may have lacked exposure to previous H1N1 epidemics and in the context of freely available antivirals and latterly a specific pandemic vaccine, we were fortunate to suffer only three deaths in our study population. In addition, a large number of our almost entirely European Caucasian patients benefited from the application of NIV - this was not seen in either Canada or Australasia. Our low mortality rate prevented assessment of the STSS scores' discriminatory potential with regard to H1N1.

Although the STSS scale has only 4 points (as opposed to a maximum of 24 on the SOFA scale), the STSS discriminated between outcomes equally well in this study, and crude data analysis suggest that it may be better. The ROC areas for the STSS score which related to both secondary outcomes of ICU admission and need for MV demonstrated both higher values and better fit than the SOFA score values, despite our small sample size.

Although the Ontario staged triage protocol has not been evaluated as a predictor of health-care resource usage, our study suggests that SOFA's utility in this respect was fair for ICU admission (AUC 0.77) and good for MV (AUC 0.87). Part of the Ontario's recommended remit was to 'identify at an early stage those patients not responding to treatment and therefore likely to have a poor outcome ... once treatment and care start ... by formal periodic assessments to determine whether ... they are not responding to treatment or are deteriorating despite treatment, and so further treatment should be withheld in favor of symptom relief' [19]. Ferreira and colleagues [10] note that length of stay (LOS) was not related to outcome prediction when using SOFA and that the mean SOFA score had a better prognostic value than the other SOFA-derived variables; that is, patients who presented with a limited degree of organ dysfunction and had a long stay could still have a high likelihood of survival.

An increase in SOFA score in the first 48 hours is associated with 53% risk of death and a mean LOS 12.4 days. However, Khan and colleagues [12] showed 63% survival in their SOFA defined poor prognosis group with H1N1 with a mean LOS of 11 (range of 3 to 17) days. This presents a problem if we assume that the DOH intended that the triage tool be applied to limit those 12.4 days by early palliation. If we also consider the published risk of death following delta SOFA reduction (23%) to the risk of death with delta SOFA increase (37%) of Khan and colleagues, this significantly narrows the difference in risk between those who would possibly die or possibly survive (14%). Hick and colleagues [22] suggest that this level of difference would not be enough to confidently reallocate ventilator resources.

The SOFA score was designed for ICU admission, and the STSS system was designed for hospital admission. Their use as the organ dysfunction component within a triage prioritization tool designed for use at all levels of health-care delivery from community to critical care obviates any concerns about the difference between secondary and tertiary triage measures. Eighty-four percent of the patients were transferred to the ICU less than 24 hours after their admission and we would therefore have expected an equivalent or better performance from the SOFA score. The three patients who had their scores calculated at a time distant from their admission to the ICU had SOFA scores ranging from 1 to 5 and an STSS score of 2. The patient with the SOFA score of 1 was admitted to the ICU after 2 days as an in-patient but did not survive; he was readmitted to the hospital with pneumonia after being discharged home (but still H1N1-positive) and died of multi-organ failure.

We would submit that these factors suggest that the STSS performs better in this population and overall would be a more appropriate early-assessment triage rule. Given the heterogeneity of possible events causing a mass casualty episode, no single tool can be expected to provide adequate decision-making power. Owing to the potential uncertainty arising from each individual patient's physiological response to treatment, scoring systems must be tempered by clinical decision making and viewed as indicators to assist clinical assessments and not as definitive triaging values. Owing to the unpredictable nature of any new strain of a pandemic virus, many authorities call for continuous revalidation and refinement of any triage model and its scoring systems at the point of outbreak and throughout all of its phases to thereby determine their suitability and discriminating power for use as triage prioritization tools at a multicenter/national level [8,18,19].

It is clear that, in the absence of a thoughtful approach to triage, critical care resources would be depleted within the first weeks of a pandemic. Such triage tools will also require a staged approach that needs to start with the patient in the community and to triage in a stepwise manner those who ultimately will require maximum care in a critical care unit. The STSS score is designed to be used at the front door of the hospital and may be of value in indicating patients who will require high resource utilization. In our small cohort, it appeared to perform as well as, if not better than, the SOFA score in identifying those who needed ICU care. The focus at the hospital level would be on establishing the process that will be followed at the health-care facility. This is crucial because, regardless of the origin of the decision tool, the implementation of the tool occurs at the hospital level [22]. Therefore, adequate workforce knowledge and training regarding the underlying principles of any prioritization tool are required to overcome the natural reluctance of medical staff to 'ration' their care delivery [27].

Our study is limited by its retrospective nature, size, and number of significant events. The performance of STSS scores in 'all comers' to the ED and specifically to the ICU as well as over the time course of each individual admission was not assessed, raising concerns about the validity of these scores in other critically ill patients. However, it should be remembered that the SOFA score was also initially developed, in 1996, as a sepsis-related score [7]. What our study highlights is that mandating a particular scoring system may not be the best approach. Perhaps considering different tools in the early phase, including perhaps one employing a disease-specific scoring system, and quickly assessing their utility may be the most pragmatic approach until the clinical course and pathophysiology of the particular influenza variant become apparent.

## Conclusions

In summary, it would appear that the four groupings of the STSS score, despite being underpowered because of a small sample size, number of deaths, and percentage of those mechanical ventilated, 'accurately' risk-stratify patients in this cohort according to their risk of death and predict the likelihood of admission to critical care and the requirement for mechanical ventilation in line with the derivation population. The fact that the STSS score is measured at a single point in time, its accuracy, and its easily collected component variables commend it as an alternative reproducible system to facilitate the triage and treatment of patients in any future influenza pandemic. Further analysis should include a prospective evaluation of its validity as a staged protocol in a larger cohort of unselected unwell patients presenting to the ED and an assessment of its morbidity and mortality prediction in different populations once those patients have been admitted to the ICU.

## Key messages

• The Simple Triage Scoring System (STSS) score is easier to calculate and accurately predicts critical care resource usage (admission to the intensive care unit and requirement for mechanical ventilation) in H1N1 with initial hospital presentation parameters.

• Further analysis of the STSS score as a predictor of mortality in this cohort of patients should be investigated.

• The STSS score should be considered an alternative triage tool in future epidemics.

## Abbreviations

AUC: area under the curve; COPD: chronic obstructive pulmonary disease; CXR: chest x-ray; DOH: Department of Health; ECMO: extracorporeal membrane oxygenation; ED: emergency department; FiO_2_: fraction of inspired oxygen; ICU: intensive care unit; LOS: length of stay; MV: mechanical ventilation; NIV: noninvasive ventilation; ROC: receiver operating characteristic; SOFA: Sepsis-related Organ Failure Assessment; STSS: Simple Triage Scoring System.

## Competing interests

The authors declare that they have no competing interests.

## Authors' contributions

Both authors participated in the formulation and design of this study, performed the literature search, and abstracted the data. KAA wrote the first draft of the manuscript, which was then revised by RC. Both authors read and approved the final manuscript.
